# Supplementary material for: Gender Differences in Attachment Anxiety and Avoidance and Their Association with Psychotherapy Use—Examining Students from a German University
Source: Behav Sci (Basel). 2022 Jun 22;12(7):204. doi: 10.3390/bs12070204 (PMC9312160; doi:10.3390/bs12070204)
Supplement: Supplementary file 1 [file behavsci-12-00204-s001.zip › behavsci-1765453-supplementary.pdf]

Gender differences in attachment anxiety and avoidance and their association with psychotherapy use – Examining students from a German university

Weber, R.[1], Eggenberger, L.[2], Stosch, C.[3], Walther, A.[2,*]

[1] University of Cologne, Faculty of Medicine and University Hospital Cologne, Clinic and Polyclinic for Psychosomatics and Psychotherapy, Germany
[2] Clinical Psychology and Psychotherapy, University of Zurich, Zurich, Switzerland
[3] Student Deans Office, Medical Faculty, University of Cologne, Germany
[*] Correspondence

Supplementary material

Content

S-Table 1: *Logistic Regression Models predicting current Psychotherapy Use* *page 2*

S-Table 2: *Logistic Regression Models predicting Intention to Start Psychotherapy* *page 3*

S-Table 3: *Gender Differences in Psychotherapy Use for Syndromic Subgroups* *page 4*

S-Figure 1: *Odds Ratios for current Psychotherapy Use for Male Students* *page 6*

S-Figure 2: *Odds Ratios for current Psychotherapy Use for Feale Students* *page 7*

S-Figure 3: *Odds Ratios for Intention to Start Psychotherapy for Male Students* *page 8*

S-Figure 4: *Odds Ratios for Intention to Start Psychotherapy for Female Students* *page 9*

S-Figure 5: *Significant Moderations* *page 10*

S-Figure 6: Sample flow diagram leading to final sample *page 11*

Exploratory moderation analysis: Results *page 12*

S-Table 1. *Logistic Regression Models predicting current Psychotherapy Use*

Subsample	Predictor	β *(SE)*	*p*	*p (corr.)*	R^2	AIC
Total	Attachment Anxiety	0.39 *(0.04)*	**< .001*****	**< .001*****	.13	2619
	Attachment Avoidance	0.07 *(0.04)*	.135	.942		
Men	Attachment Anxiety	0.47 *(0.08)*	**< .001*****	**< .001*****	.16	538
	Attachment Avoidance	0.19 *(0.10)*	**.046***	.370		
Women	Attachment Anxiety	0.37 *(0.04)*	**< .001*****	**< .001*****	.12	2082
	Attachment Avoidance	0.05 *(0.05)*	.328	1		
Any syndrome	Attachment Anxiety	0.32 *(0.04)*	**< .001*****	**< .001*****	.13	1801
	Attachment Avoidance	0.06 *(0.05)*	.202	1		
Any depressive syndrome	Attachment Anxiety	0.27 *(0.05)*	**< .001*****	**< .001*****	.10	1199
	Attachment Avoidance	0.07 *(0.06)*	.218	1		
Any anxiety syndrome	Attachment Anxiety	0.28 *(0.07)*	**< .001*****	**.001****	.09	554
	Attachment Avoidance	0.03 *(0.08)*	.761	1		
Alcohol use syndrome	Attachment Anxiety	0.36 *(0.08)*	**< .001*****	**< .001*****	.14	487
	Attachment Avoidance	0.20 *(0.10)*	**.039***	0.349		
Somatoform syndrome	Attachment Anxiety	0.24 *(0.07)*	**< .001*****	**.003****	.11	757
	Attachment Avoidance	-0.04 *(0.08)*	.650	1		
Any eating disorder	Attachment Anxiety	0.49 *(0.10)*	**< .001*****	**< .001*****	.28	373
	Attachment Avoidance	-0.08 *(0.11)*	0.471	1		

Note. β = estimated regression coefficient, *SE* = standard error, *p* = *p*-value, R^2 = Nagelkerke's maximum-likelihood estimation for pseudo *R*-squared, AIC = Akaike Information Criterion. All calculations were controlled for age and nationality. *corr.* = *p*-values were adjusted for multiple testing using the Holm-Bonferroni method.

* = $p < .05$, ** = $p < .01$, *** = $p < .001$

S-Table 2. *Logistic Regression Models predicting Intention to Start Psychotherapy*

Subsample	Predictor	β *(SE)*	*p*	*p (corr.)*	R^2	AIC
Total	Attachment Anxiety<br>Attachment Avoidance	0.36 (0.02)<br>0.14 (0.03)	**< .001*****<br>**< .001*****	**< .001*****<br>**< .001*****	.15	5330
Men	Attachment Anxiety<br>Attachment Avoidance	0.44 (0.05)<br>0.14 (0.06)	**< .001*****<br>**.017***	**< .001*****<br>.087	.18	1162
Women	Attachment Anxiety<br>Attachment Avoidance	0.32 (0.03)<br>0.16 (0.03)	**< .001*****<br>**< .001*****	**< .001*****<br>**< .001*****	.14	4150
Any syndrome	Attachment Anxiety<br>Attachment Avoidance	0.29 (0.03)<br>0.13 (0.03)	**< .001*****<br>**< .001*****	**< .001*****<br>**< .001*****	.14	3499
Any depressive syndrome	Attachment Anxiety<br>Attachment Avoidance	0.27 (0.04)<br>0.15 (0.04)	**< .001*****<br>**< .001*****	**< .001*****<br>**.002****	.13	2198
Any anxiety syndrome	Attachment Anxiety<br>Attachment Avoidance	0.23 (0.06)<br>0.13 (0.06)	**< .001*****<br>**.047***	**< .001*****<br>.147	.11	804
Alcohol use syndrome	Attachment Anxiety<br>Attachment Avoidance	0.36 (0.05)<br>0.13 (0.06)	**< .001*****<br>**.039***	**< .001*****<br>.147	.14	1098
Somatoform syndrome	Attachment Anxiety<br>Attachment Avoidance	0.25 (0.04)<br>0.1 (0.05)	**< .001*****<br>**.037***	**< .001*****<br>.147	.12	1502
Any eating disorder	Attachment Anxiety<br>Attachment Avoidance	0.23 (0.06)<br>0.11 (0.07)	**< .001*****<br>.119	**< .001*****<br>.147	.26	663

Note. β = estimated regression coefficient, *SE* = standard error, *p* = *p*-value, R^2 = Nagelkerke's maximum-likelihood estimation for pseudo *R*-squared, AIC = Akaike Information Criterion. All calculations were controlled for age and nationality. *corr.* = *p*-values were adjusted for multiple testing using the Holm-Bonferroni method.

* = $p < .05$, ** = $p < .01$, *** = $p < .001$

S-Table 3. *Gender Differences regarding Psychotherapy Use in Different Syndromic Subgroups*

A) Current Psychotherapy Use in Different Syndromic Subgroups

Syndrome	Men, *N (%)*	Women, *N (%)*	*z (df)*	*p*	*p* (corr.)
Any syndrome	61 (9.0)	251 (11.5)	-2.33 (2851)	**.020***	.241
Any depressive	43 (11.0)	169 (12.8)	-1.27 (1705)	.202	1
Minor depression	12 (7.7)	54 (9.9)	-0.88 (698)	.381	1
Major depression	31 (13.1)	115 (14.9)	-0.91 (1002)	.362	1
Any anxiety	19 (17.4)	91 (18.0)	-0.3 (609)	.763	1
Anxiety-panic	7 (33.3)	23 (24.7)	0.22 (109)	.824	1
Anxiety-generalized	15 (14.9)	78 (17.4)	-0.67 (544)	.506	1
Any eating	13 (10.6)	64 (14.1)	-1.67 (929)	.094	1
Eating-binge	9 (9.6)	45 (13)	-1.35 (1152)	.178	1
Eating-bulimia	4 (13.8)	19 (17.8)	-1.36 (571)	.175	1
Somatoform syndrome	11 (8.6)	118 (11.5)	-0.59 (131)	.553	1
Alcohol use syndrome	23 (6.8)	54 (9.1)	-1.33 (436)	.184	1

B) Intention to Start Psychotherapy in Different Syndromic Subgroups

Syndrome	Men, *N (%)*	Women, *N (%)*	*z (df)*	*p*	*p* (corr.)
Any syndrome	217 (32.0)	868 (39.9)	-3.72 (2851)	**<.001*****	**.002****
Any depressive	166 (42.3)	630 (47.8)	-1.89 (1705)	.059	.474
Minor depression	52 (33.3)	185 (33.8)	-0.15 (698)	.880	1
Major depression	114 (48.3)	445 (57.7)	-2.42 (1002)	**.015***	.155
Any anxiety	60 (55.0)	285 (56.4)	-0.25 (609)	.802	1
Anxiety-panic	14 (66.7)	62 (66.7)	0.34 (109)	.734	1
Anxiety-generalized	56 (55.4)	248 (55.4)	-0.02 (544)	.988	1
Any eating	35 (28.5)	182 (40.2)	-2.77 (929)	**.006****	.062
Eating-binge	25 (26.6)	125 (36.0)	-0.81 (1152)	.421	1
Eating-bulimia	10 (34.5)	58 (54.2)	-2.34 (571)	**.019***	.172

Somatoform syndrome	55 (43.0)	477 (46.4)	-1.64 (131)	.101	.608
Alcohol use syndrome	94 (27.6)	214 (36.0)	-1.79 (436)	.073	.512

Note. N = number of participants, *df* = degrees of freedom, *p* = *p*-value, (corr.) = adjusted for multiple testing using the Holm method.

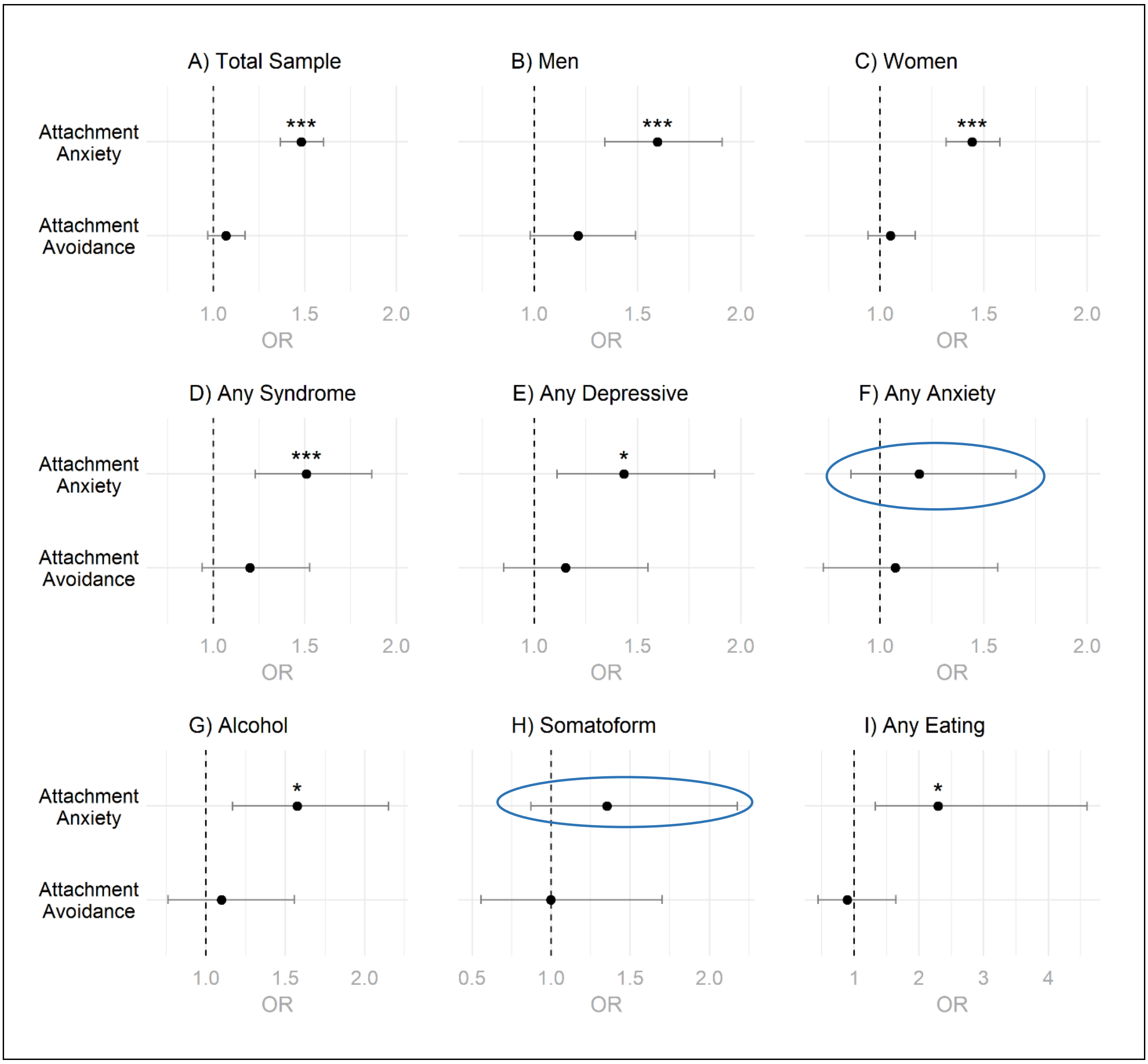

S-Figure 1. *Odds Ratios for current Psychotherapy Use for Male Students*

Note. OR = Odds Ratio. The error bars indicate a 95% confidence interval and the blue highlightings mark significant changes as compared to the total sample (Figure 4). All calculations were controlled for age and nationality. *p*-values were adjusted for multiple testing using the Holm-Bonferroni method.
* = *p* < .05, ** = *p* < .01, *** = *p* < .001

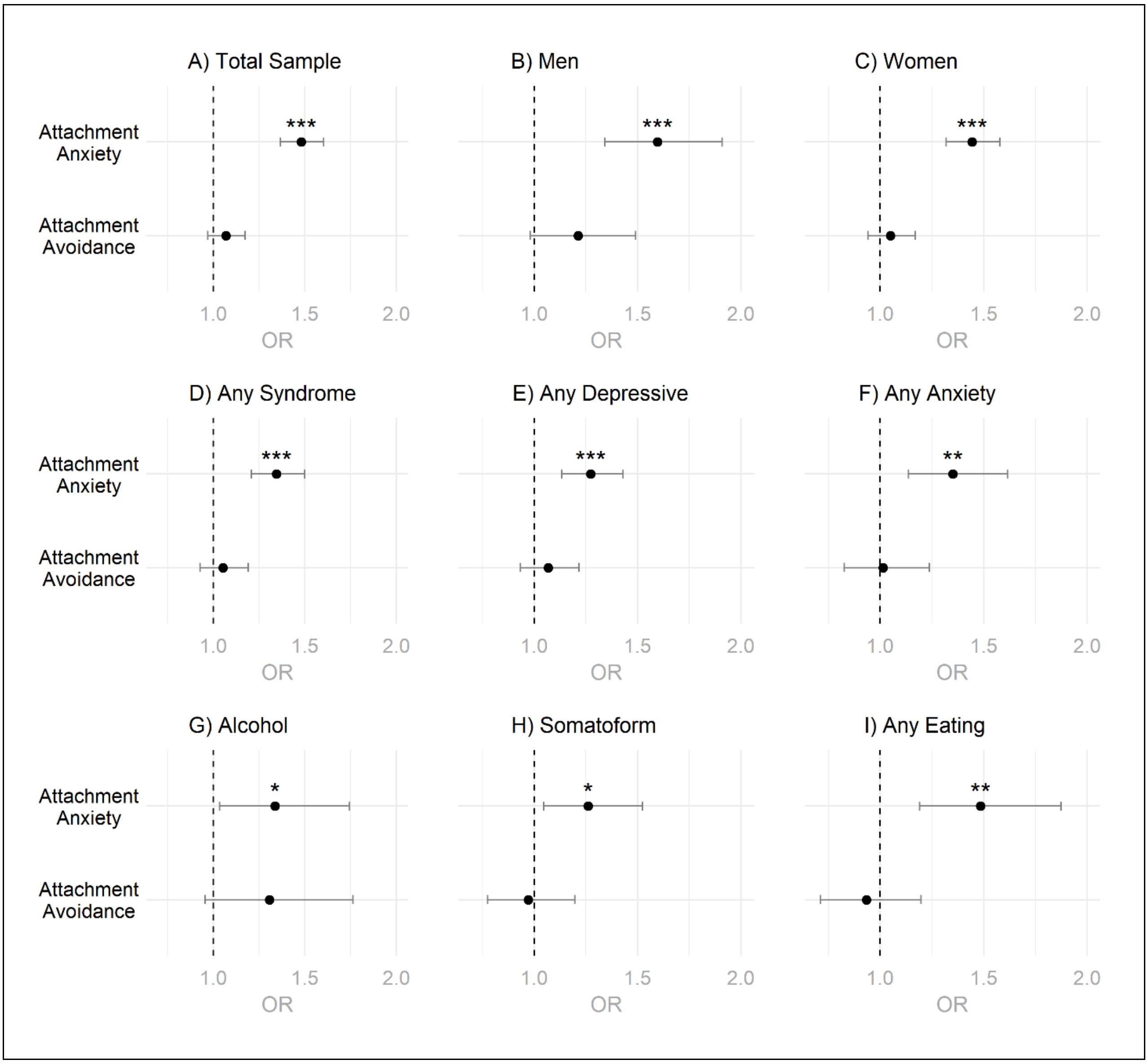

S-Figure 2. *Odds Ratios for current Psychotherapy Use for Female Students*

Note. OR = Odds Ratio. The error bars indicate a 95% confidence interval. There were no significant changes compared to the total sample (Figure 4). All calculations were controlled for age and nationality. *p*-values were adjusted for multiple testing using the Holm-Bonferroni method.
* = $p < .05$, ** = $p < .01$, *** = $p < .001$

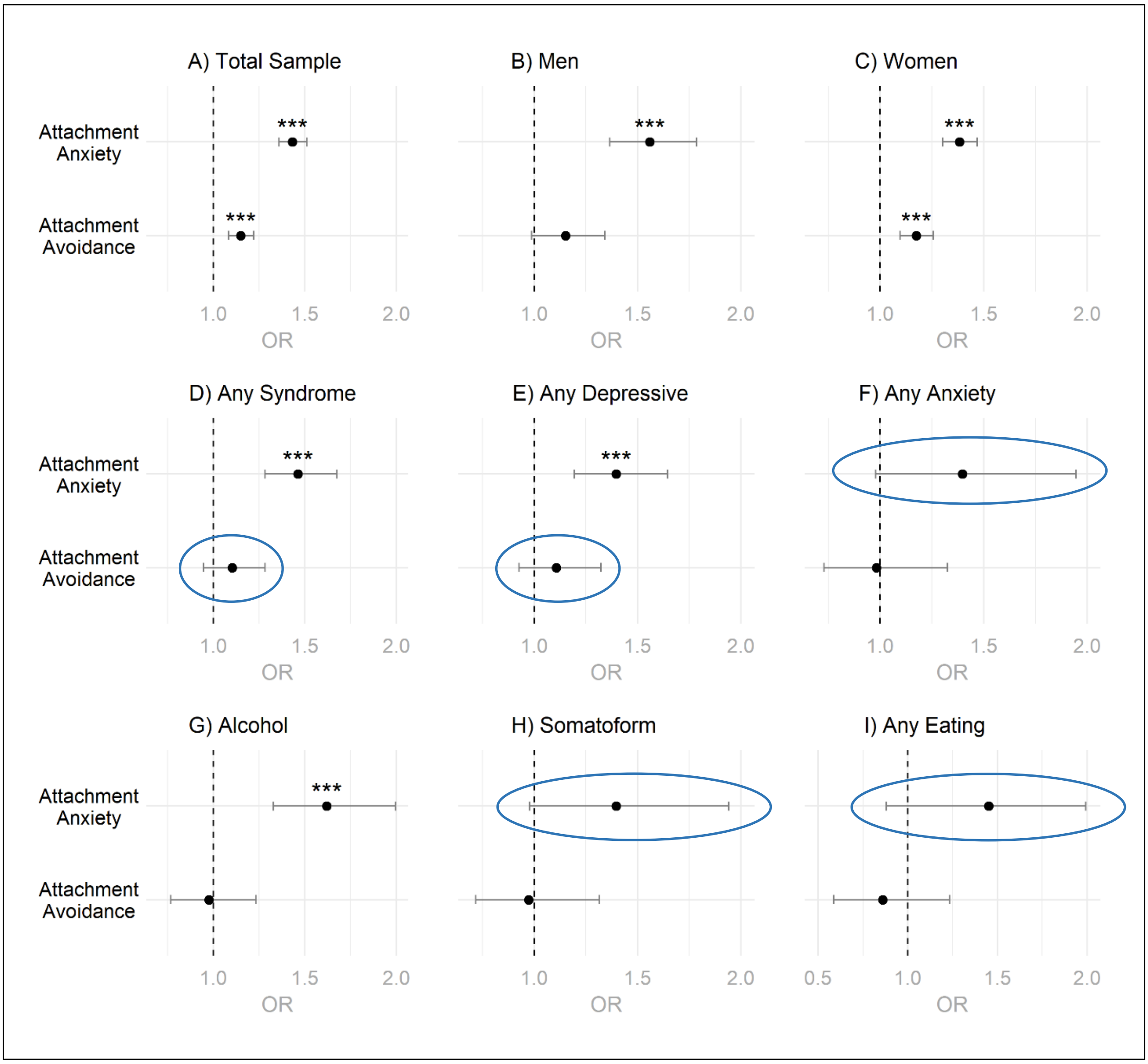

S-Figure 3. *Odds Ratios for Intention to Start Psychotherapy for Male Students*

Note. OR = Odds Ratio. The error bars indicate a 95% confidence interval and the blue highlightings mark significant changes as compared to the total sample (Figure 5). All calculations were controlled for age and nationality. *p*-values were adjusted for multiple testing using the Holm-Bonferroni method.

$^{*} = p < .05$, $^{**} = p < .01$, $^{***} = p < .001$

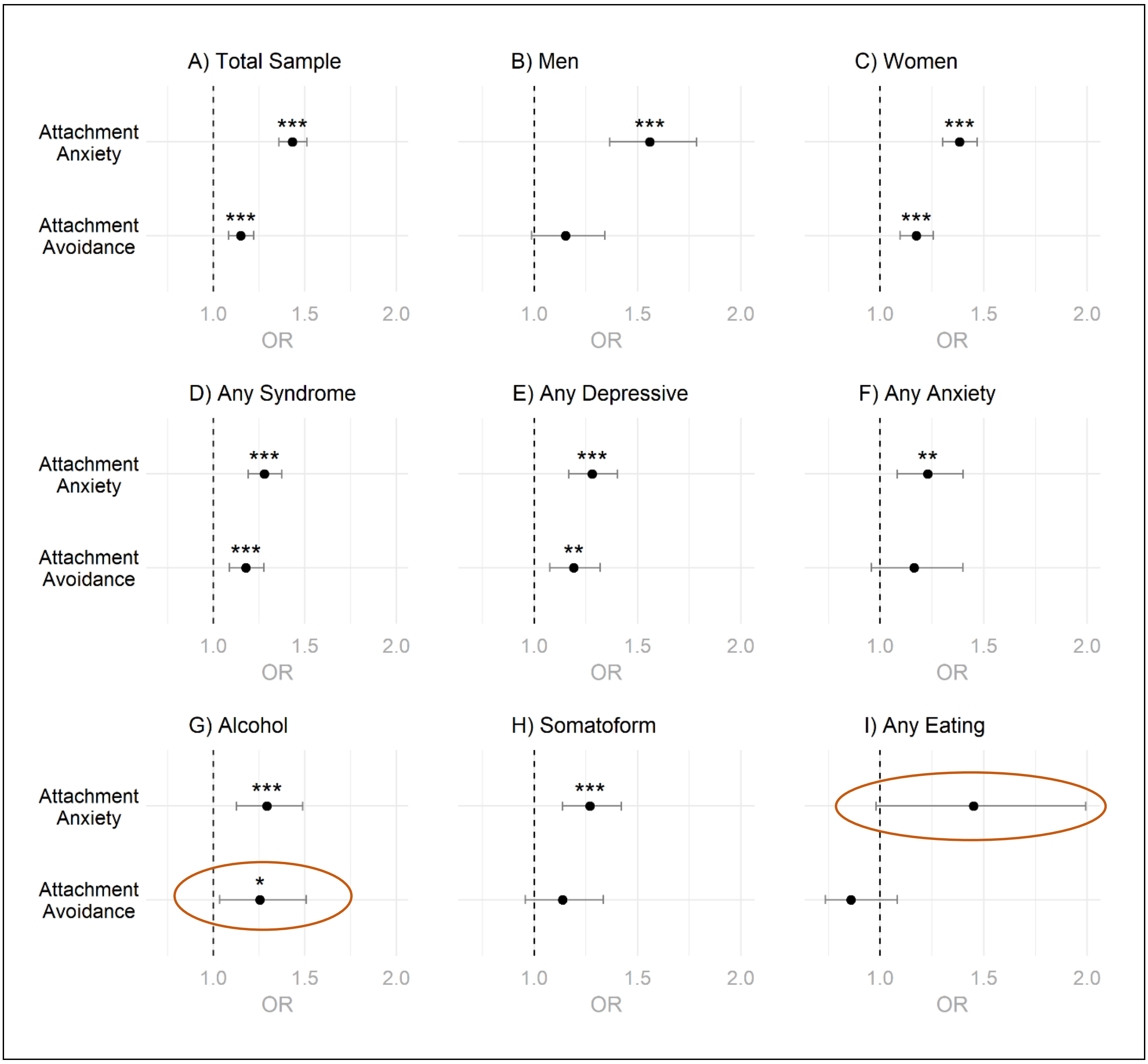

S-Figure 4. *Odds Ratios for Intention to Start Psychotherapy for Female Students*

Note. OR = Odds Ratio. The error bars indicate a 95% confidence interval and the orange highlightings mark significant changes as compared to the total sample (Figure 5). All calculations were controlled for age and nationality. *p*-values were adjusted for multiple testing using the Holm-Bonferroni method.
* = $p < .05$, ** = $p < .01$, *** = $p < .001$

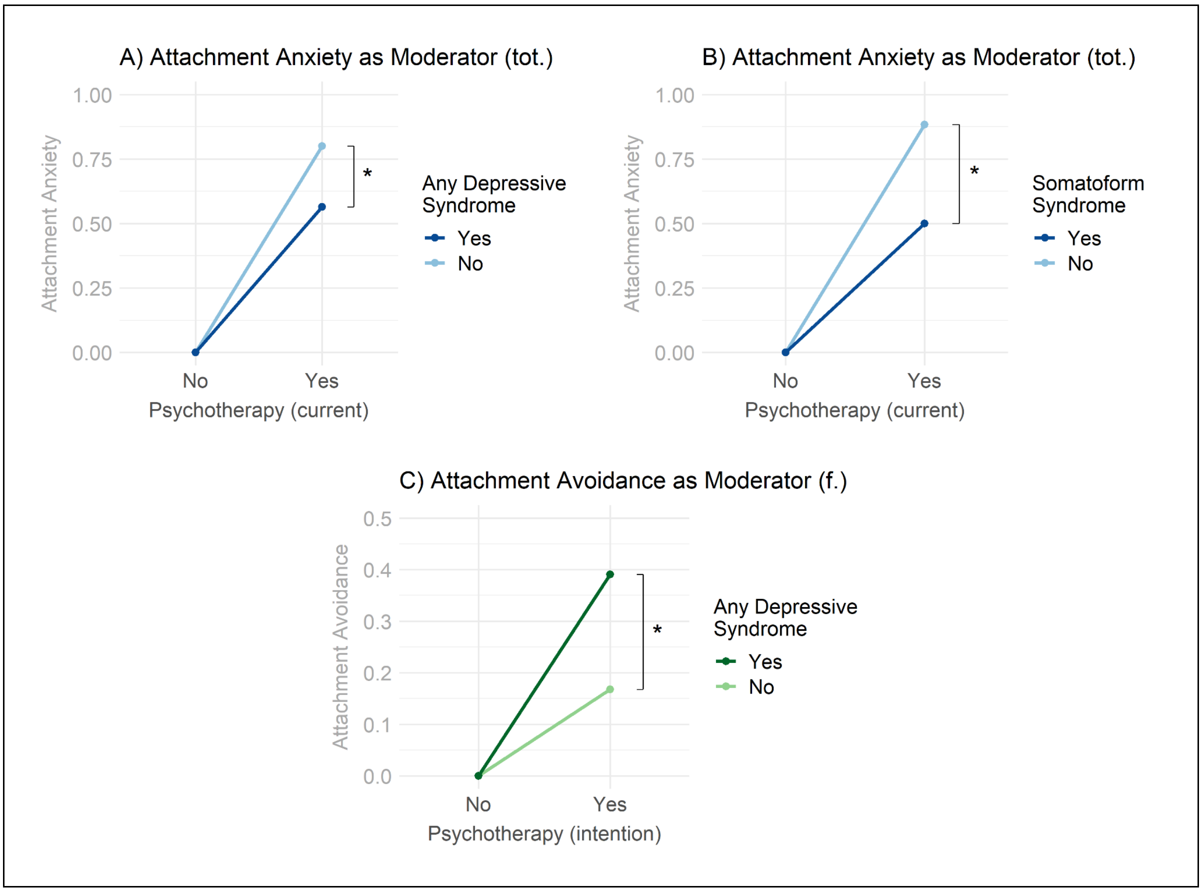

S-Figure 5. *Significant Interactions regarding Attachment Dimensions, Psychiatric Syndromes, and Psychotherapy Use*

Note. tot. = total sample, *f.* = female subsample, *p*-values were not adjusted for multiple testing.
* = $p < .05$

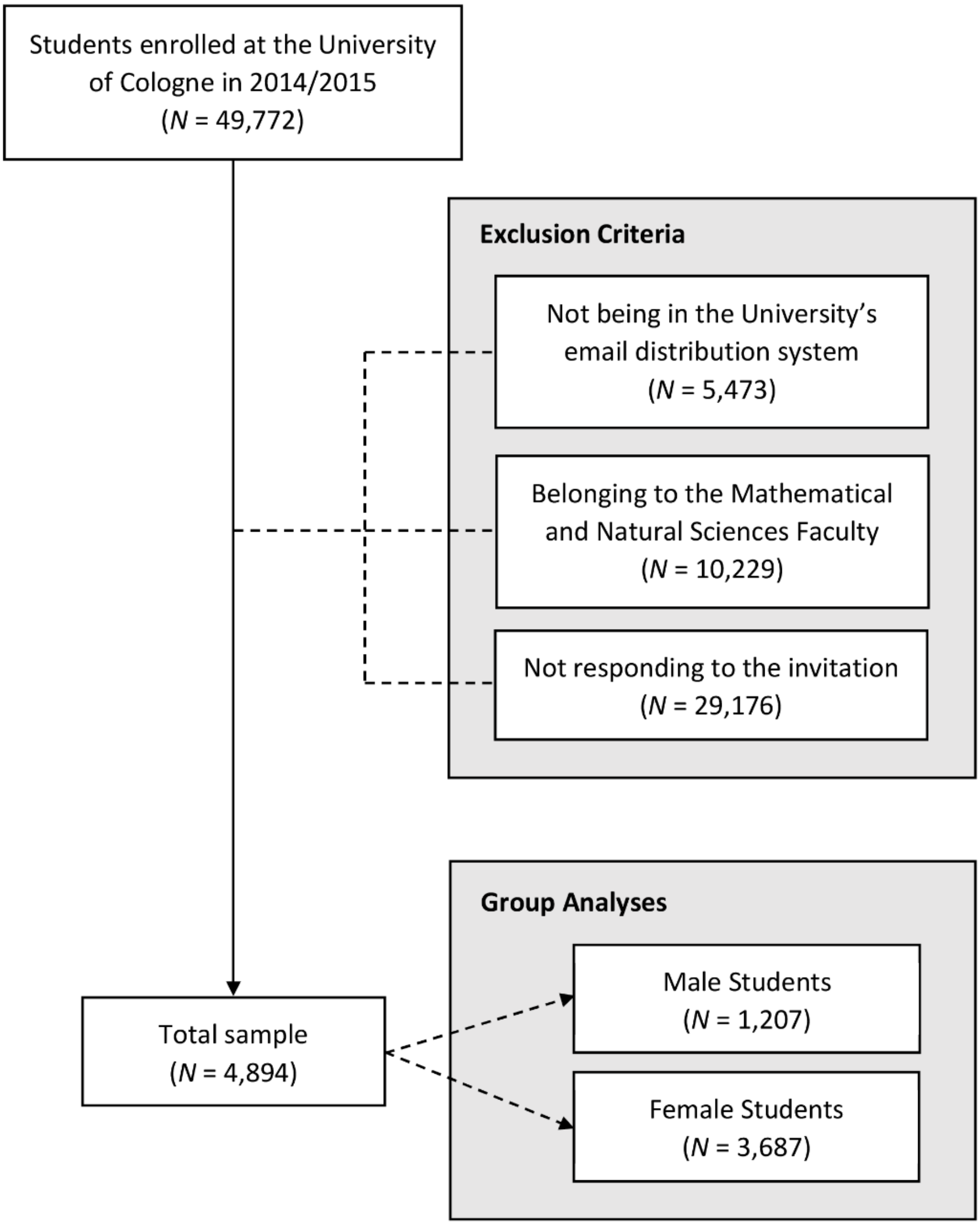

S-Figure 6. Sample flow leading to final sample
Note. *N* = number of participants

Exploratory moderation analysis – Results

Explorative moderation analyses were further conducted to detect possible moderating effects of attachment orientation on the association of individual syndromes and psychotherapy use. Moderation analyses were conducted using Hayes' (2013) regression-based approach to test for possible moderating effects of attachment orientation on the association between the likelihood to use or the intention to start psychotherapy and the positive screening for different psychiatric syndromes. Subsequently described significant moderation effects did not survive correction for multiple testing, suggesting that the results should be considered preliminary findings providing a basis for future research and a call for replication.

In a first part, where current psychotherapy use was the outcome, the only two significant interaction effects were observed for attachment anxiety on the association between current psychotherapy use and any depressive syndrome (z[4697] = 2.35, p = .019, Nagelkerke R^2 = .136., AIC = 2601.6) and somatoform syndrome (z[4697] = -2.51, p = .012, Nagelkerke R^2 = .135, AIC = 2603.1), respectively (S-Figure 5A – 5B). Both interactions indicate that students screened negative for any depressive or somatoform syndrome are more likely to currently use psychotherapy when exhibiting higher attachment anxiety as compared to students screened negative for any depressive or somatoform syndrome who exhibit lower attachment anxiety. The same interactions were also be observed in the female subsample (any depressive syndrome: z[3542] = 2.10, p = .036, Nagelkerke R^2 = .129, AIC = 2069.3; somatoform syndrome: z[3542] = -2.13, p = .033, Nagelkerke R^2 = .129, AIC = 2069.9), but not in the male subsample (any depressive syndrome: z[1147] = 0.87, p = .387, Nagelkerke R^2 = .168, AIC = 538.5; somatoform syndrome: z[1147] = -0.50, p = .611, Nagelkerke R^2 = .166, AIC = 539.8).

In a second part, where the intention to start psychotherapy was used as an outcome, the only significant interaction (S-Figure 5C) could be observed in the female subsample for attachment avoidance on the association between the intention to start psychotherapy and any depressive syndrome (z[3542] = -1.99, p = .047, Nagelkerke R^2 = .191, AIC = 4003.1). This interaction indicates that female students screened positive for any depressive syndrome would be more likely to intend starting psychotherapy when exhibiting higher attachment avoidance as compared to female students screened positive for any depressive syndrome with lower attachment avoidance. Conversely, these results were not found in the total sample (z[4697] = -1.95, p = .051, Nagelkerke R^2 = .191, AIC = 5164.1) nor in the male subsample (z[1147] = -0.11, p = .910, Nagelkerke R^2 = .210, AIC = 1141.9).

Hayes, A. F. (2013). *Introduction to mediation, moderation, and conditional process analysis: A regression-based approach*. New York, NY: Guilford Press.
